# Risk factors of prognosis for spontaneous cerebellar hemorrhage: a systematic review and meta-analysis

**DOI:** 10.1007/s00701-024-06174-z

**Published:** 2024-07-10

**Authors:** Junbin Shu, Wei Wang, Ruyong Ye, Yonggang Zhou, Jianfeng Tong, Xiaobo Li, Xiaojun Lv, Guangliang Zhang, Feng Xu, Jing Zhang

**Affiliations:** Department of Neurosurgery, The First People’s Hospital of Yongkang City, Yongkang, China

**Keywords:** Spontaneous cerebellar hemorrhage, Prognosis, Risk factors, Meta-analysis

## Abstract

**Background:**

The most deadly type of spontaneous intracerebral hemorrhage is spontaneous cerebellar hemorrhage (SCH). The purpose of this meta-analysis was to investigate risk factors for prognosis in SCH patients to provide a basis for taking preventive and therapeutic measures.

**Methods:**

Seven electronic databases were searched from inception to May 2023 for randomized controlled trial, cohort study, case control study and cross-sectional study on prognosis of spontaneous cerebellar hemorrhage. The quality of the selected studies were assessed by the American Agency for Healthcare Research and Quality (AHRQ). To assess the impact of the included risk factors on the prognosis of spontaneous cerebellar hemorrhage, combined odds ratios (ORs) with matching 95% confidence intervals (CIs) were combined.

**Results:**

Eight studies were included, including 539 participants. And a total of 31 potentially associated risk factors were identified. Ultimately, 6 risk factors were included in the meta-analysis after assessing. The factors supported by moderate evidence include the hydrocephalus (OR = 4.3, 95% CI: 2.33 to 7.91) and drug-induced coagulopathy (OR = 2.74, 95% CI: 1.23 to 6.09). The factors supported by limited evidence include the intraventricular bleeding(OR = 1.86, 95% CI: 1.13 to 3.07) and hematoma size>3 cm(OR = 3.18, 95% CI: 1.87 to 5.39). Meta-analysis revealed no association between hypertension, diabetes mellitus and SCH prognosis.

**Conclusion:**

The current meta-analysis revealed obvious risk factors for prognosis in spontaneous cerebellar hemorrhage patients, including hydrocephalus, drug-induced coagulopathy, intraventricular bleeding and hematoma size>3 cm.

**Supplementary Information:**

The online version contains supplementary material available at 10.1007/s00701-024-06174-z.

## Introduction

Although it accounts for only 5 to 13% of all spontaneous intracerebral hemorrhage [28, 29], spontaneous cerebellar hemorrhage (SCH) is arguably the most deadly type of the condition (30-day mortality rate ranging from 30 to 50% [7, 9]) due to its peculiar neurological location close to the brainstem. Therefore, early detection of prognostic risk factors is crucial for early therapy and prevention. In SCH patients, prognostic factors for poor outcome or early mortality have been reported, including larger hematoma volumes or diameter, a Glasgow Coma Scale (GCS) ≤ 8, and imaging findings that show the presence of hydrocephalus, intraventricular hemorrhage (IVH), brainstem compression, or basal cistern obligation [17, 30]. Due to the various methodologies, it is challenging to confirm the risk factors for SCH prognosis in several research. Thus, in order to establish a foundation for the implementation of preventative and therapeutic measures, this meta-analysis was carried out to investigate the risk factors for prognosis in SCH patients.

## Methods

The study was conducted according to the Preferred Reporting Items for Systematic Reviews and Meta-Analysis (PRISMA) [16]. A protocol was created prospectively, outlining the precise goals, selection criteria, technique for judging study quality, clinical outcomes, and statistical methods. The PRISMA checklist was followed while reporting the study (Supplementary Table 1).

## Sources and search strategy

Search was conducted from inception to May 2023, in PubMed, Embase, Web of Science, the Cochrane Library, China National Knowledge Information Database (CNKI), WanFang Database, and Chinese Scientific Journal Database (VIP). These seven databases were inclusive of most of the possible articles related to our research topic, regardless of language and reported risk factors for postoperative SCH; there were no language or publication data constraints. The search strategy followed MeSH terms and all related free search terms:(((((((Cerebellums) OR (Corpus Cerebelli)) OR (cerebellar)) OR (Parencephalon)) OR (parencephalon)) OR ("Cerebellum"[Mesh])) AND (((((((((((((((((Outcome, Treatment) OR (Patient-Relevant Outcome)) OR (Outcome, Patient-Relevant)) OR (Outcomes, Patient-Relevant)) OR (Patient Relevant Outcome)) OR (Patient-Relevant Outcomes)) OR (Clinical Effectiveness)) OR (Effectiveness, Clinical)) OR (Treatment Effectiveness)) OR (Effectiveness, Treatment)) OR (Rehabilitation Outcome)) OR (Outcome, Rehabilitation)) OR (Treatment Efficacy)) OR (Efficacy, Treatment)) OR (Clinical Efficacy)) OR (Efficacy, Clinical)) OR ("Treatment Outcome"[Mesh]))) AND (((Hemorrhages) OR (Bleeding)) OR ("Hemorrhage"[Mesh])).

## Inclusion and exclusion criteria

Studies were independently screened for inclusion on the basis of title and abstract by two reviewers (W.W. and R.-Y.Y.). The study was included and reevaluated in the second round of inclusion in the event of a dispute. Full-text screening was used for the second round of inclusion, and disagreements between reviewers were settled through discussion or consultation with a third reviewer (Y.-G.Z.). The studies in this meta-analysis matched the following criteria: (1)The studies had to be conducted on patients aged ≥ 16 years diagnosed with SCH. (2)The study had to be a randomized controlled trial, cohort study, case control study or cross-sectional study. (3) Studies that had original, unambiguous odds ratio (OR) data and a 95% confidence interval (CI) that could be calculated or extrapolated. The following studies were excluded:(1) Studies without data or without factor analysis. (2) conference abstracts, conference papers, reviews and meta-analyses. (3) republished studies. We did not search gray literature or any unpublished materials.

## Data extraction

One researcher (J.-F.T.) extracted data from the included studies using a pre-made worksheet. First author, publication year, geographic location, study design, sample size, mean age, relevant risk factors and quality score were all taken from each study. The correctness and completeness of the extracted data were reviewed by another researcher (X.-B.L.). We discussed disagreements and looked to the main report for guidance.

## Quality assessment

The same two reviewers (J.-F.T. and X.-B.L.) independently assessed cross-sectional studies using the American Agency for Healthcare Research and Quality (AHRQ) [15]. There were 11 questions in the AHRQ that answered “yes”, “no” or “unclear”. If the answer is “no” or “unclear” the score is 0, and if the answer is “yes”, the score is 1, a score of 1–3 indicates low quality, 4–7 suggests medium quality, and 8–11 indicates good quality.

## Strength of evidence

The current evidence scales were used for assessment [11, 26] and were defined as follows in order to identify the amount of evidence for each risk factor and based on the caliber of the studies: (1) strong evidence: results are based on three or more studies, at least two of which were high-quality homogeneous studies or several high-quality studies synthesized. (2) moderate evidence: results that are statistically significant when one high-quality study is combined with one or more studies of a moderate or low quality. (3) limited evidence: either a single high-quality study or a combination of several moderate- or low-quality studies produced the results. (4) very limited evidence or the no evidence: significantly pooled results from multiple studies where heterogeneity findings were unrelated to quality.

## Statistical analysis

Forest plots were used for demonstrating the combined results among the same factors, studies were categorized according to the type of risk factor. We only conducted a meta-analysis of the risk factors evaluated in at least three distinct studies in order to guarantee the reliability of the pooled effect estimates size. Data from two or fewer studies or factors with different results were presented in tables without summary analysis. The effect of risk factors on the prognosis for SCH was assessed using pooled ORs and related 95% CIs. The software was utilized for conversion if there were no OR values. Heterogeneity across all included studies was assessed and quantified using I^2^ statistics [12]. Heterogeneity increases with a higher I^2^ value. The I^2^ values of 25%, 50% and 75% represent the low, moderate, and high degrees of heterogeneity respectively [6]. By omitting each study from the meta-analysis, sensitivity analysis was carried out for results with high heterogeneity to determine the stability of the conclusions [1, 10]. Stata software (Stata version 16.0, College Station, Texas, USA) was used for all analyses.

## Results

### Study selection

**Fig. 1:**
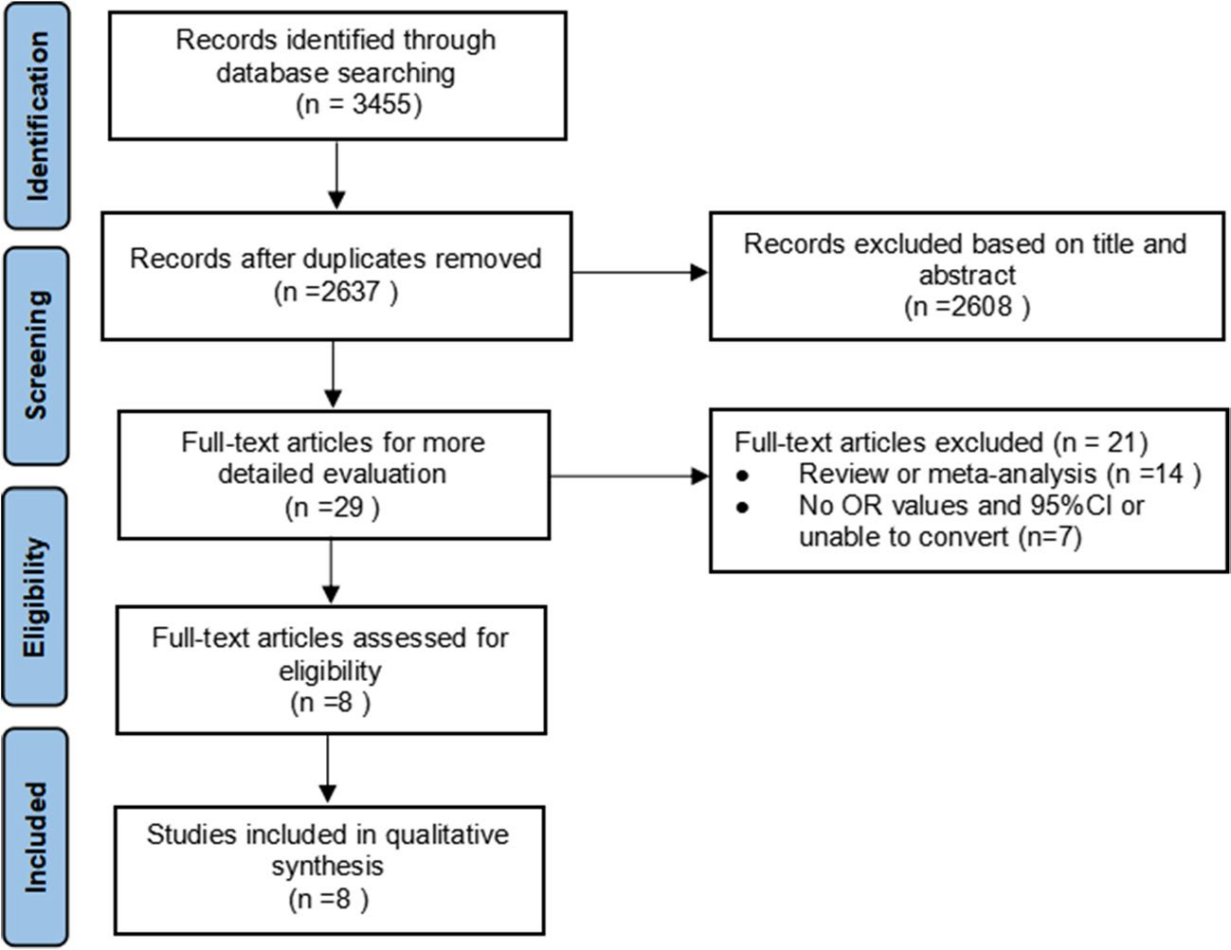
Flowchart of the search process for the articles

A total of 3455 related studies were yielded by searching seven electronic databases, of which 2637 retained after deleting duplicates and 2608 were disregarded after reading the titles and abstracts. Upon applying the exclusion criteria, 21 studies were removed. The remaining 8 studies matched the inclusion criteria. A PRISMA flow chart illustrates the study selection process and reasons for study exclusion (Fig. 1).

### Study characteristics and quality assessment

The basic characteristics of the included studies and quality evaluation results were summarized in Table 1. Eight cross-sectional studies were included [3, 17, 18, 28–30, 32, 33], of these, 1 were rated as high quality, 7 were rated as medium quality.

**Table 1:** Characteristics of the studies included in the meta-analysis

First author	Publication year	Geographic region	Study design	Sample size	Mean age	Relevant risk factors	Quality score
Cohen ZR	2002	Israel	Cross-sectional study	30	NR	1–4	6
Dammann P	2011	Germany	Cross-sectional study	57	64.4	1、3–12	4
Monayer S	2021	Israel	Cross-sectional study	53	68.52 ± 10.67	5、6、16–19	7
Zhao SZ	2022	China	Cross-sectional study	121	61.08 ± 11.62	1、5、10、11、13、15、19、24、26	7
Yang T	2020	China	Cross-sectional study	49	56.9 ± 17.65	8	7
Shen J	2021	China	Cross-sectional study	62	67.39 ± 10.21	4、6、11、13、15、16、24、26–33	8
Matsukawa H	2012	Japan	Cross-sectional study	53	67(Median age)	4、10	7
Satop J	2017	Finland	Cross-sectional study	114	68.3	1、4、5、7、8、10、11、19、22–25	7

Relevant risk factors: 1:hematoma size>3 cm; 2:Glasgow scale<13; 3:Pyramidal signs; 4:Hydrocephalus; 5:Hypertension; 6:Drug-induced coagulopathy; 7:Compression of the 4th ventricle; 8:Brain stem compression; 9:Tight posterior fossa; 10:Intraventricular bleeding; 11:Age; 12:Initial level of consciousness; 13:Gender; 14: Craniectomy; 15:Vascular risk factors (any); 16: BMI ≥ 30; 17:Weight ≥ 100 kg; 18:Dyslipidemia; 19:Diabetes mellitus; 20:Atrial fibrillation; 21:Liver diseases; 22: hematoma volume; 23:Quadrigeminal cistern obliteration; 24:External ventricular drainage; 25:Daily alcohol consumption; 26:Early surgery; 27:Duration of surgery; 28:Black hole sign; 29:Island sign; 30:Swirl sign; 31:Mixed density on CT scan. NR: not reported

### Risk factors of prognosis in spontaneous cerebellar hemorrhage

**Fig. 2:**
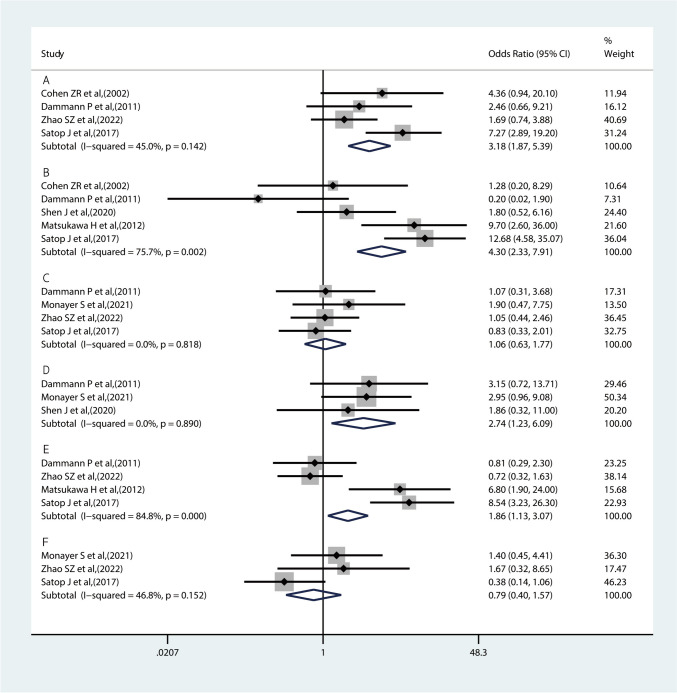
Meta-analysis of risk factors for prognosis in spontaneous cerebellar hemorrhage (**A**: hematoma size>3 cm, **B**: Hydrocephalus, **C**: Hypertension, **D**: Drug-induced coagulopathy, **E**: Intraventricular bleeding, **F**: Diabetes mellitus,)

Overall, 31 potential risk factors were extracted from 8 studies, including hematoma size>3 cm, Glasgow scale>13, pyramidal signs, hydrocephalus, hypertension, drug-induced coagulopathy, compression of the 4th ventricle, brain stem compression, tight posterior fossa, intraventricular bleeding, age, initial level of consciousness, gender, craniectomy, vascular risk factors, BMI ≥ 30, Weight ≥ 100 kg, dyslipidemia, diabetes mellitus, atrial fibrillation, liver diseases, hematoma volume, quadrigeminal cistern obliteration, external ventricular drainage, daily alcohol consumption, early surgery, duration of surgery, black hole sign, island sign, swirl sign and mixed density on CT scan. There were 6 risk factors, including hematoma size>3 cm, hydrocephalus, hypertension, drug-induced coagulopathy, intraventricular bleeding and diabetes mellitus that met the criteria for inclusion in the meta-analysis (Supplementary Table 2). The combined results are presented in Fig. 2.

#### *Hematoma size*>*3 cm*

A total of four studies [3, 5, 19, 33] reported the effect of hematoma size>3 cm on the prognostic outcome in spontaneous cerebellar hemorrhage patients, involving 322 patients. Our results showed that hematoma size>3 cm is a risk factor for the prognosis of spontaneous cerebellar hemorrhage (OR = 3.18, 95% CI: 1.87 to 5.39, *P* = 0.142). Heterogeneity was considered moderately insignificant (I^2^ = 45.0%).

#### Hydrocephalus

Five studies [3, 5, 14, 19, 20] reported a relationship between hydrocephalus and the prognosis of spontaneous cerebellar hemorrhage. Our pooled results demonstrated that hydrocephalus was closely related to the occurrence of poor outcome on spontaneous cerebellar hemorrhage (OR = 4.3, 95% CI: 2.33to 7.91, *P*=0.002). However, sensitivity analysis, subgroup analysis and meta-regression were carried out because of the significant heterogeneity among the studies (I^2^ = 75.7%), and the adjusted results were in line with the initial results (Supplementary Fig. 1A to 3A).

#### Hypertension

Four studies [3, 18, 19, 33] met our criteria for meta-analysis, and the combined results suggested no association with hypertension to poor outcome in SCH (OR = 1.06, 95% CI: 0.63 to 1.77, *P*=0.818). The heterogeneity was minimal (I^2^ = 0%).

#### Drug-induced coagulopathy

The drug-induced coagulopathy was reported in 3 studies [3, 18, 20] with minimal heterogeneity (OR = 2.74, 95% CI: 1.23 to 6.09, I^2^ = 0.0%, *P* = 0.890). The combined results depicted that a higher probability of poor outcome on spontaneous hemorrhage occurred.

#### Intraventricular bleeding

Four studies [3, 14, 19, 33] were pooled to identify the relationship between intraventricular bleeding and prognostic outcome in spontaneous cerebellar hemorrhage patients. The results of the combined meta-analysis showed that the intraventricular bleeding significantly increased the risk of developing poor outcome in spontaneous cerebellar hemorrhage patients with a OR of 1.86 (95% CI: 1.13 to 3.07, I^2^ = 84.8%, *P* < 0.00001). Due to the high heterogeneity, sensitivity analysis, subgroup analysis and meta-regression were carried out, and the adjusted results were in line with the initial results (Supplementary Fig. 1B to 3B).

#### Diabetes mellitus

Three studies [18, 19, 33] reported the impact of diabetes mellitus on poor outcome in spontaneous cerebellar hemorrhage. The combined results demonstrated no association with diabetes mellitus to poor outcome in SCH (OR = 0.79, 95% CI: 0.40 to 1.57). The heterogeneity was moderate (I^2^ = 46.8%, *P* = 0.152).

## Egger’s test

The correlation between the effect estimates and their variances was investigated using the Egger's test for each risk factor, with a *P* value of < 0.05 indicating a statistically significant difference [8].The results of Egger's test did not reveal any statistical support for publication bias (Table 2).

**Table 2:** The results of publication bias

Risk factors	Egger’s test	
	*t* value	*p* value
Hematoma size>3 cm	‒0.34	0.765
Hydrocephalus	2.64	0.077
Hypertension	‒1.70	0.231
Drug-induced coagulopathy	1.05	0.484
Intraventricular bleeding	‒1.47	0.280
Diabetes mellitus	‒1.01	0.497

## Discussion

This study investigated the prognosis risk factors in patients with spontaneous cerebellar hemorrhage and performed a meta-analysis of 6 risk factors, comprising 8 papers. Meta-analysis revealed that 4 risk factors were related to poor outcome in spontaneous cerebellar hemorrhage patients. Due to the limited number of studies, we did not enroll the left 25 risk factors into meta-analysis. To date, no other published meta-analysis has shown relevant prognostic factors for spontaneous cerebellar hemorrhage.

We found moderate certainty evidence that SCH patients who had hydrocephalus were more likely to experience a poor outcome. The likely reason is that the posterior fossa is a small compartment with little extra room to handle the mass effect. Thus, obstructive hydrocephalus and brainstem compression may result from the hematoma and its accompanying swelling, and in extreme situations, which may lead to early mortality [4]. Another significant risk factor for the prognosis of spontaneous cerebellar hemorrhage is drug-induced coagulopathy. According to a recent study, approximately 12 to 20% of patients presenting with intracerebral hemorrhage are taking oral anticoagulants, which is associated with hematoma expansion, and increased risk of poor outcome and death [23]. A small randomized clinical trail did demonstrate reversal of drug-induced coagulopathy could reduce hematoma expansion, with a trend toward improved patient outcome [24]. According to numerous studies [13, 21], intraventricular bleeding is an independent predictor of a worse prognosis in spontaneous cerebellar hemorrhage, which is consistent with our findings. The hematoma rupture into ventricular system, forming acute obstructive hydrocephalus, causing a severe increase in intracranial pressure, inducing cerebral hernia, and ultimately leading to cardiac and respiratory arrest [27]. Thus, early surgical treatment is recommended to quickly remove cerebellar hematoma and relieve mechanical compression, but the timing of the surgery is still controversial [22]. In addition, Hematoma size>3 cm has also been shown to predict a poor outcome in spontaneous cerebellar hemorrhage. It is commonly acknowledged that hematoma size is an independent risk factor for both short- and long-term poor prognosis in patients with SCH [13, 25, 31]. However, our study revealed that hypertension had no significant relationship with the risk of poor outcome in spontaneous cerebellar hemorrhage. In contrast, Monayer S et al. found that hypertension is an independent risk factor for the poor prognosis of SCH [18]. Thus, more studies are needed to further verify these findings. Diabetes mellitus have frequently been identified as a risk factor for poor outcome in SCH [31], but in the current review, diabetes mellitus had no effect on the prognosis of SCH. As we expected, possibly due to the nonstandardization of how blood glucose was measured and differences in the baseline characteristics of the study cohorts. Therefore, the role of diabetes mellitus in poor prognosis of SCH patients remains to be elucidated.

Our findings elucidate a significant association between hydrocephalus, drug-induced coagulopathy, intraventricular bleeding and hematoma size>3 cm and the prognosis of spontaneous cerebellar hemorrhage, which advances the current understanding in this field.Unlike previous isolated studies, our comprehensive approach integrates data across a broader spectrum of research, offering a more consolidated view of the potential prognostic markers for spontaneous cerebellar hemorrhage.

## Strengthens and limitations

To the best of our knowledge, this is the first systematic review and meta-analysis of risk factors for prognosis in spontaneous cerebellar hemorrhage patients. It provides the most comprehensive evidence of risk factors for SCH prognosis, including hydrocephalus, drug-induced coagulopathy, intraventricular bleeding and hematoma size>3 cm.

Our review also suffered from a few limitations. We were unable to do a meta-analysis because many factors were only mentioned in a single article, and a lack of stratified effect estimates by GCS < 6 or other factors. Uncompleted data reporting, such as many studies only provided p-values [30] or OR without 95% CI [2], was another limiting factor that prevented us from performing a meta-analysis of the majority of variables. And the heterogeneity were high in the meta-analysis of intraventricular bleeding and hydrocephalus. Although sensitivity analysis, subgroup analysis and meta-regression were performed, the source of heterogeneity was not well defined. Finally, most included studies were cross-sectional, so no causal relationship between exposure factors and outcomes was established, and recall bias is likely to exist.

## Conclusion

The current meta-analysis revealed obvious risk factors for prognosis in spontaneous cerebellar hemorrhage patients, including hydrocephalus, drug-induced coagulopathy, intraventricular bleeding and hematoma size>3 cm. Our review found moderate certainty evidence that the hydrocephalus and drug-induced coagulopathy had a higher probability of poor outcome among SCH patients. These risk factors may help clinicians identify high-risk patients to improve prognosis.

## Supplementary Information

Below is the link to the electronic supplementary material.

Supplementary file1 (DOCX 1632 KB)

Supplementary file2 (DOCX 73 KB)

Supplementary file3 (DOCX 114 KB)

Supplementary file4 (DOCX 28 KB)

Supplementary file5 (DOCX 19 KB)

Supplementary file6 (DOCX 12 KB)

## Data Availability

All data are included in this manuscript.
