# Supplementary material for: Risk factors of prognosis for spontaneous cerebellar hemorrhage: a systematic review and meta-analysis
Source: Acta Neurochir (Wien). 2024 Jul 10;166(1):291. doi: 10.1007/s00701-024-06174-z (PMC11236867; doi:10.1007/s00701-024-06174-z)
Supplement: Supplementary file 1 — Supplementary file1 (DOCX 1632 KB) [file 701_2024_6174_MOESM1_ESM.docx]

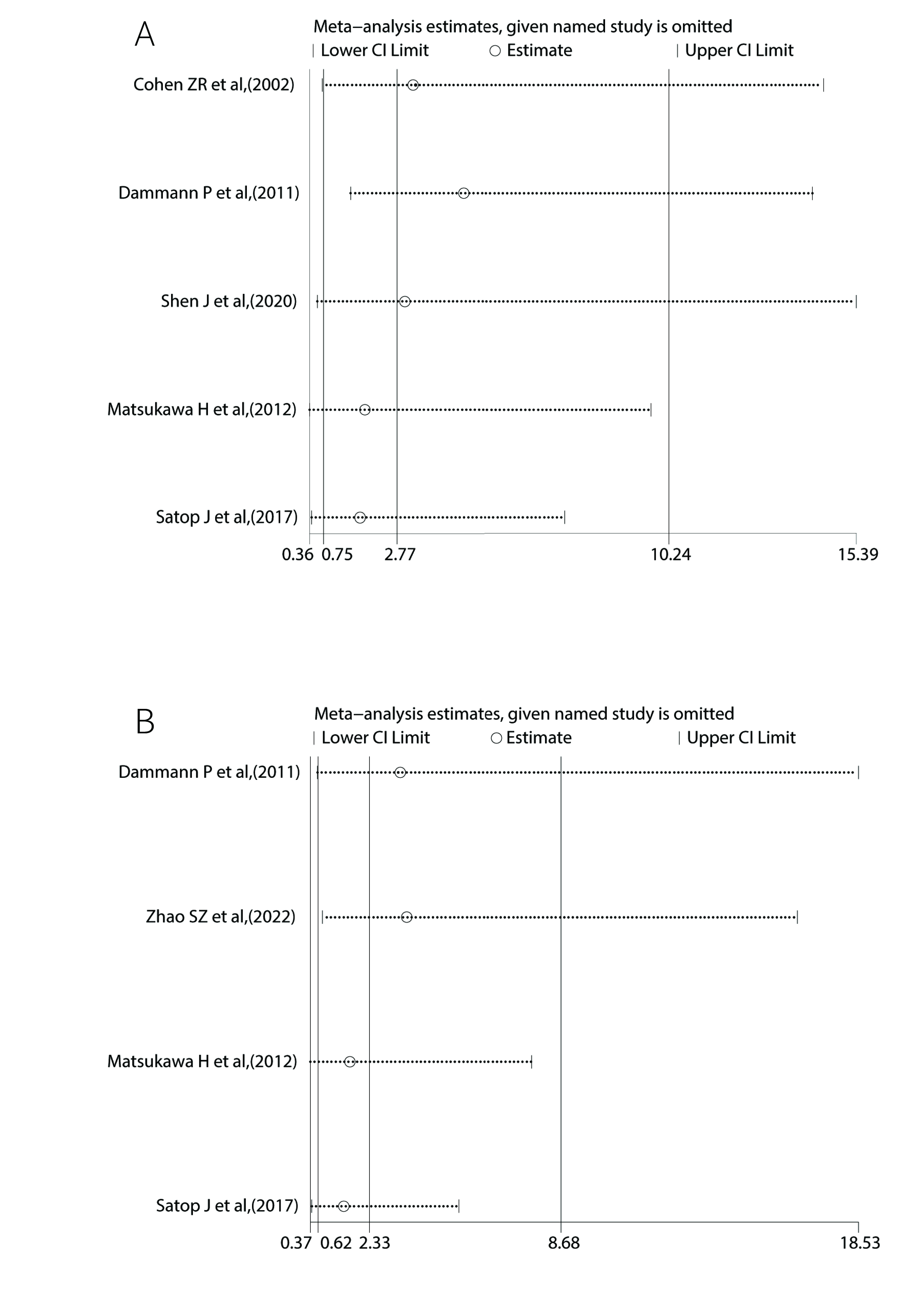


**Supplementary figure 1.** Sensitivity analysis of of risk factors for prognosis in spontaneous cerebellar hemorrhage (A:Hydrocephalus, B:Intraventricular bleeding)
