# Supplementary material for: Risk factors of prognosis for spontaneous cerebellar hemorrhage: a systematic review and meta-analysis
Source: Acta Neurochir (Wien). 2024 Jul 10;166(1):291. doi: 10.1007/s00701-024-06174-z (PMC11236867; doi:10.1007/s00701-024-06174-z)
Supplement: Supplementary file 5 — Supplementary file5 (DOCX 19 KB) [file 701_2024_6174_MOESM5_ESM.docx]

| Risk factor | Studies | OR | 95%CI |
| --- | --- | --- | --- |
| Glasgow scale＜13 | Cohen ZR et al, 2002 | 1.23 | 0.89, 1.69 |
| Pyramidal signs | Cohen ZR et al, 2002 | 2.99 | 0.18, 48.9 |
|  | Dammann P et al, 2011 | 0.4783 | 0.1259, 1.8174 |
| Compression of the 4th ventricle | Dammann P et al, 2011 | 0.4310 | 0.0368, 5.0420 |
|  | Satop J et al, 2017 | 3.939 | 0.51, 51.5 |
| Brain stem compression | Dammann P et al, 2011 | 0.1635 | 0.0403, 0.6634 |
|  | Satop J et al, 2017 | 6.586 | 0.293, 3230 |
| Tight posterior fossa | Dammann P et al, 2011 | 0.1382 | 0.0273, 0.6986 |
| Age | Dammann P et al, 2011 | 0.5174 | 0.1636, 1.6365 |
|  | Zhao SZ et al, 2022 | 1.058 | 1.020, 1.097 |
|  | Shen J et al, 2020 | 1.200 | 0.147, 9.786 |
|  | Satop J et al, 2017 | 0.625 | 0.134, 2.46 |
| Initial level of consciousness | Dammann P et al, 2011 | 15.8125 | 4.1612, 60.0879 |
| Gender | Zhao SZ et al, 2022 | 1.111 | 0.520, 2.376 |
|  | Shen J et al, 2020 | 0.208 | 0.025, 1.757 |
| Craniectomy | Zhao SZ et al, 2022 | 5.506 | 2.135, 14.198 |
|  | Shen J et al, 2020 | 0.343 | 0.117, 1.009 |
| Vascular risk factors(any) | Monayer S et al, 2021 | 12.86 | 1.325, 124.692 |
|  | Shen J et al, 2020 | 0.857 | 0.221, 3.320 |
| BMI≥30 | Monayer S et al, 2021 | 3.55 | 1.002, 12.639 |
| Weight≥100kg | Monayer S et al, 2021 | 3.14 | 0.627, 15.755 |
| Dyslipidemia | Monayer S et al, 2021 | 3.871 | 2.311, 6.484 |
| Atrial fibrillation | Satop J et al, 2017 | 0.876 | 0.286, 3.14 |
| Liver diseases | Satop J et al, 2017 | 0.689 | 0.155, 4.01 |
| Haematoma volume | Zhao SZ et al, 2022 | 2.071 | 0.941, 4.560 |
|  | Shen J et al, 2020 | 17.000 | 1.683, 171.703 |
|  | Satop J et al, 2017 | 4.517 | 1.87, 11.6 |
| Qsuadrigeminal cistern obliteration | Satop J et al, 2017 | 6.456 | 2.34, 20.6 |
| External ventricular drainage | Zhao SZ et al, 2022 | 4.371 | 1.355, 14.105 |
|  | Shen J et al, 2020 | 0.343 | 0.117, 1.009 |
| Daily alcohol consumption | Shen J et al, 2020 | 1.548 | 0.336, 7.130 |
| Early surgery | Shen J et al, 2020 | 3.176 | 1.003, 10.059 |
| Duration of surgery | Shen J et al, 2020 | 1.524 | 0.250, 9.295 |
| Black hole sign | Shen J et al, 2020 | 3.635 | 0.690, 19.138 |
| Island sign | Shen J et al, 2020 | 5.000 | 0.548, 45.579 |
| Swirl sign | Shen J et al, 2020 | 3.647 | 0.576, 19.033 |
| Mixed density on CT scan | Shen J et al, 2020 | 2.043 | 0.680, 6.140 |

**Supplementary table 2.** Other risk factors for prognosis of spontaneous cerebellar hemorrhage.
