# Supplementary material for: Risk factors of prognosis for spontaneous cerebellar hemorrhage: a systematic review and meta-analysis
Source: Acta Neurochir (Wien). 2024 Jul 10;166(1):291. doi: 10.1007/s00701-024-06174-z (PMC11236867; doi:10.1007/s00701-024-06174-z)
Supplement: Supplementary file 6 — Supplementary file6 (DOCX 12 KB) [file 701_2024_6174_MOESM6_ESM.docx]

**Supplementary table 3. Search strategy**.

Pubmed:

(((((((Cerebellums) OR (Corpus Cerebelli)) OR (cerebellar)) OR (Parencephalon)) OR (parencephalon)) OR ("Cerebellum"[Mesh])) AND (((((((((((((((((Outcome, Treatment) OR (Patient-Relevant Outcome)) OR (Outcome, Patient-Relevant)) OR (Outcomes, Patient-Relevant)) OR (Patient Relevant Outcome)) OR (Patient-Relevant Outcomes)) OR (Clinical Effectiveness)) OR (Effectiveness, Clinical)) OR (Treatment Effectiveness)) OR (Effectiveness, Treatment)) OR (Rehabilitation Outcome)) OR (Outcome, Rehabilitation)) OR (Treatment Efficacy)) OR (Efficacy, Treatment)) OR (Clinical Efficacy)) OR (Efficacy, Clinical)) OR ("Treatment Outcome"[Mesh]))) AND (((Hemorrhages) OR (Bleeding)) OR ("Hemorrhage"[Mesh]))

Embase:

1: 'cerebellar organisation' OR 'cerebellar organization' OR 'cerebellum'

2: 'abnormal bleeding' OR 'bleeding complication' OR 'blood effusion' OR 'blood loss' OR 'capillary bleeding' OR 'haemorrhage' OR 'haemorrhage model' OR 'haemorrhagic activity' OR 'hemorrhage' OR 'hemorrhage model' OR 'hemorrhagia' OR 'hemorrhagic activity' OR 'spontaneous haemorrhage' OR 'spontaneous hemorrhage' OR 'bleeding'

3: 'health care outcome and process assessment' OR 'healthcare outcome and process assessment' OR 'medical futility' OR 'outcome and process assessment (health care)' OR 'outcome and process assessment, health care' OR 'outcome management' OR 'patient outcome' OR 'therapeutic outcome' OR 'therapy outcome' OR 'treatment outcome'

4: 1 AND 2 AND 3

Web of Science:

(((AB=(TS=(Cerebellums OR Corpus Cerebelli OR Cerebella OR Parencephalon OR Parencephalons OR Cerebellum))) AND AB=(TS=(Hemorrhages OR Bleeding OR Hemorrhage))) AND AB=(TS=(Patient Relevant Outcome OR Clinical Effectiveness OR Treatment Effectiveness OR Rehabilitation Outcome OR Treatment Efficacy OR Clinical Efficacy OR Treatment Outcome)))

the Cochrane Library:

1: MeSH descriptor: [Cerebellum] explode all trees

2: (cerebellum):ti,ab,kw OR (Cerebellums):ti,ab,kw OR (Corpus Cerebelli):ti,ab,kw OR (Cerebella):ti,ab,kw OR (parencephalon):ti,ab,kw

3: (parencephalons):ti,ab,kw

4: 1 OR 2 OR 3

5: MeSH descriptor: [Hemorrhage] explode all trees

6: (hemorrhage):ti,ab,kw OR (hemorrhages):ti,ab,kw OR (bleeding):ti,ab,kw

7: 4 OR 5

8: MeSH descriptor: [Treatment Outcome] explode all trees

9: (treatment outcome):ti,ab,kw OR (Outcome, Treatment):ti,ab,kw OR (Patient-Relevant Outcome):ti,ab,kw OR (Outcome, Patient-Relevant):ti,ab,kw OR (Outcomes, Patient-Relevant):ti,ab,kw

10: (Patient Relevant Outcome):ti,ab,kw OR (Patient-Relevant Outcomes):ti,ab,kw OR (Clinical Effectiveness):ti,ab,kw OR (Effectiveness, Clinical):ti,ab,kw OR (Treatment Effectiveness):ti,ab,kw

11: (Effectiveness, Treatment):ti,ab,kw OR (Rehabilitation Outcome):ti,ab,kw OR (Outcome, Rehabilitation):ti,ab,kw OR (Treatment Efficacy):ti,ab,kw OR (Efficacy, Treatment):ti,ab,kw

12: (Clinical Efficacy):ti,ab,kw OR (Efficacy, Clinical):ti,ab,kw

13: 8 OR 9 OR 10 OR 11

14: 4 AND 7 AND 13

China National Knowledge Information Database (CNKI):

(SU % '小脑') AND (SU % '出血') AND (SU % '预后')

WanFang Database：

(主题:(小脑)) * (主题:(出血)) * (主题:(预后))

Chinese Scientific Journal Database (VIP)：

M=小脑 AND M=出血 AND M=预后
